# Supplementary material for: Lean thinking by integrating with discrete event simulation and design of experiments: an emergency department expansion
Source: PeerJ Comput Sci. 2020 Aug 10;6:e284. doi: 10.7717/peerj-cs.284 (PMC7924453; doi:10.7717/peerj-cs.284)
Supplement: Supplemental Information 1 [file peerj-cs-06-284-s001.docx]

**Supplementary Material 1 – DoE – Locals**

Table 1 – CCF design used in DOE

| **Run Order** | **SSU** | **Bed** | **Vertical Area** | **Mental** | **LOS** | **Treated** | **Triage- Bed** | **Prep-SSU** |
| --- | --- | --- | --- | --- | --- | --- | --- | --- |
| 1 | 10 | 45 | 10 | 3 | 863.52 | 66.49% | 153.74 | 1405.83 |
| 2 | 30 | 45 | 10 | 3 | 447.21 | 93.59% | 34.96 | 167.27 |
| 3 | 10 | 53 | 10 | 3 | 506.70 | 73.84% | 80.49 | 1389.94 |
| 4 | 30 | 53 | 10 | 3 | 438.76 | 94.75% | 33.42 | 171.80 |
| 5 | 10 | 45 | 15 | 3 | 660.73 | 62.33% | 100.46 | 1174.36 |
| 6 | 30 | 45 | 15 | 3 | 425.55 | 93.50% | 27.04 | 165.20 |
| 7 | 10 | 53 | 15 | 3 | 546.22 | 67.47% | 81.28 | 1128.15 |
| 8 | 30 | 53 | 15 | 3 | 391.47 | 95.05% | 24.77 | 27.61 |
| 9 | 10 | 45 | 10 | 5 | 766.96 | 65.53% | 152.32 | 1154.10 |
| 10 | 30 | 45 | 10 | 5 | 421.17 | 87.28% | 16.57 | 69.47 |
| 11 | 10 | 53 | 10 | 5 | 523.93 | 72.88% | 106.83 | 1196.70 |
| 12 | 30 | 53 | 10 | 5 | 405.53 | 96.08% | 22.08 | 77.05 |
| 13 | 10 | 45 | 15 | 5 | 746.69 | 64.75% | 152.07 | 991.85 |
| 14 | 30 | 45 | 15 | 5 | 416.55 | 94.68% | 17.58 | 94.76 |
| 15 | 10 | 53 | 15 | 5 | 610.52 | 71.40% | 129.73 | 1253.52 |
| 16 | 30 | 53 | 15 | 5 | 398.02 | 94.94% | 12.24 | 55.55 |
| 17 | 10 | 49 | 13 | 4 | 694.52 | 65.98% | 144.02 | 1322.74 |
| 18 | 30 | 49 | 13 | 4 | 425.69 | 93.43% | 23.60 | 169.26 |
| 19 | 20 | 45 | 13 | 4 | 420.56 | 92.04% | 35.47 | 333.24 |
| 20 | 20 | 53 | 13 | 4 | 392.94 | 84.56% | 19.44 | 420.10 |
| 21 | 20 | 49 | 10 | 4 | 417.84 | 90.96% | 39.32 | 509.29 |
| 22 | 20 | 49 | 15 | 4 | 432.67 | 91.83% | 37.14 | 487.92 |
| 23 | 20 | 49 | 13 | 3 | 406.94 | 89.97% | 42.32 | 432.82 |
| 24 | 20 | 49 | 13 | 5 | 408.50 | 92.48% | 30.83 | 390.85 |
| 25 | 20 | 49 | 13 | 4 | 414.36 | 83.58% | 30.50 | 376.31 |
| 26 | 20 | 49 | 13 | 4 | 413.24 | 83.74% | 31.47 | 376.31 |
| 27 | 20 | 49 | 13 | 4 | 420.71 | 86.96% | 36.16 | 465.04 |
| 28 | 20 | 49 | 13 | 4 | 400.08 | 80.91% | 25.39 | 377.10 |
| 29 | 20 | 49 | 13 | 4 | 424.55 | 84.89% | 37.14 | 458.50 |
| 30 | 20 | 49 | 13 | 4 | 427.01 | 85.41% | 38.41 | 461.39 |
| 31 | 20 | 49 | 13 | 4 | 423.15 | 85.96% | 36.67 | 462.98 |

**Supplementary Material 2 – DoE shifts – Triage Nurse**

Table 2 – DOE design used in Triage Nurse

| **Run Order** | **07:00 - 19:00** | **09:00 - 21:00** | **11:00 - 23:00** | **19:00 -07:00** | **LOS** | **Treated** | **Triage-Bed** | **Prep-SSU** |
| --- | --- | --- | --- | --- | --- | --- | --- | --- |
| 1 | 1 | 0 | 1 | 1 | 481.46 | 91.72% | 19.40 | 73.60 |
| 2 | 2 | 0 | 1 | 1 | 434.80 | 95.52% | 16.46 | 144.28 |
| 3 | 1 | 1 | 1 | 1 | 449.88 | 95.47% | 19.85 | 80.19 |
| 4 | 2 | 1 | 1 | 1 | 432.68 | 93.30% | 21.37 | 52.55 |
| 5 | 1 | 0 | 2 | 1 | 456.78 | 94.95% | 13.72 | 155.16 |
| 6 | 2 | 0 | 2 | 1 | 469.03 | 87.10% | 32.54 | 167.03 |
| 7 | 1 | 1 | 2 | 1 | 434.36 | 95.84% | 12.79 | 87.07 |
| 8 | 2 | 1 | 2 | 1 | 443.27 | 92.88% | 21.74 | 188.52 |
| 9 | 1 | 0 | 1 | 2 | 449.97 | 96.77% | 8.20 | 33.05 |
| 10 | 2 | 0 | 1 | 2 | 439.69 | 95.46% | 21.09 | 115.10 |
| 11 | 1 | 1 | 1 | 2 | 461.03 | 95.97% | 18.81 | 98.59 |
| 12 | 2 | 1 | 1 | 2 | 450.20 | 96.49% | 13.30 | 131.81 |
| 13 | 1 | 0 | 2 | 2 | 456.48 | 96.10% | 14.93 | 79.79 |
| 14 | 2 | 0 | 2 | 2 | 525.13 | 86.27% | 40.55 | 221.33 |
| 15 | 1 | 1 | 2 | 2 | 448.00 | 96.03% | 17.95 | 118.95 |
| 16 | 2 | 1 | 2 | 2 | 440.11 | 87.91% | 11.88 | 145.85 |

**Supplementary Material 3 – DoE shifts – ED Nurse Team 1**

Table 3 – DOE design used in ED Nurse Team 1

| **Run Order** | **07:00 - 19:00** | **09:00 - 21:00** | **11:00 - 23:00** | **19:00 -07:00** | **LOS** | **Treated** | **Triage-Bed** | **Prep-SSU** |
| --- | --- | --- | --- | --- | --- | --- | --- | --- |
| 1 | 4 | 0 | 1 | 4 | 468.63 | 95.34% | 17.28 | 114.76 |
| 2 | 8 | 0 | 1 | 4 | 512.19 | 94.11% | 32.46 | 343.59 |
| 3 | 4 | 2 | 1 | 4 | 498.46 | 95.22% | 30.74 | 157.70 |
| 4 | 8 | 2 | 1 | 4 | 529.78 | 95.48% | 41.03 | 134.02 |
| 5 | 4 | 0 | 2 | 4 | 477.21 | 95.59% | 17.38 | 114.33 |
| 6 | 8 | 0 | 2 | 4 | 462.24 | 94.99% | 14.55 | 89.45 |
| 7 | 4 | 2 | 2 | 4 | 447.41 | 95.75% | 10.68 | 109.22 |
| 8 | 8 | 2 | 2 | 4 | 467.34 | 95.29% | 20.09 | 75.72 |
| 9 | 4 | 0 | 1 | 8 | 501.40 | 94.27% | 39.06 | 147.32 |
| 10 | 8 | 0 | 1 | 8 | 478.53 | 95.38% | 17.57 | 295.50 |
| 11 | 4 | 2 | 1 | 8 | 473.49 | 94.41% | 25.25 | 222.15 |
| 12 | 8 | 2 | 1 | 8 | 456.50 | 95.97% | 28.46 | 58.14 |
| 13 | 4 | 0 | 2 | 8 | 482.28 | 95.26% | 31.62 | 136.99 |
| 14 | 8 | 0 | 2 | 8 | 457.81 | 95.56% | 14.34 | 85.23 |
| 15 | 4 | 2 | 2 | 8 | 461.77 | 95.69% | 21.79 | 49.34 |
| 16 | 8 | 2 | 2 | 8 | 464.56 | 95.29% | 15.75 | 140.53 |
| 17 | 6 | 1 | 2 | 6 | 465.10 | 95.85% | 17.57 | 106.31 |
| 18 | 6 | 1 | 2 | 6 | 475.39 | 95.82% | 22.18 | 144.38 |

**Supplementary Material 4 – DoE shifts – ED Nurse Team 2**

Table 4 – DOE design used in ED Nurse Team 2

| **Run Order** | **07:00 - 19:00** | **09:00 - 21:00** | **11:00 - 23:00** | **19:00 -07:00** | **LOS** | **Treated** | **Triage-Bed** | **Prep-SSU** |
| --- | --- | --- | --- | --- | --- | --- | --- | --- |
| 1 | 3 | 1 | 1 | 3 | 619.90 | 90.67% | 41.63 | 107.64 |
| 2 | 6 | 1 | 1 | 3 | 504.78 | 95.64% | 34.68 | 200.54 |
| 3 | 3 | 2 | 1 | 3 | 523.85 | 92.22% | 32.20 | 152.51 |
| 4 | 6 | 2 | 1 | 3 | 485.21 | 95.44% | 20.14 | 139.10 |
| 5 | 3 | 1 | 2 | 3 | 491.14 | 95.16% | 33.62 | 142.40 |
| 6 | 6 | 1 | 2 | 3 | 484.62 | 95.52% | 20.84 | 105.59 |
| 7 | 3 | 2 | 2 | 3 | 483.27 | 95.29% | 23.69 | 93.70 |
| 8 | 6 | 2 | 2 | 3 | 560.22 | 72.96% | 36.68 | 180.99 |
| 9 | 3 | 1 | 1 | 6 | 510.33 | 95.21% | 42.91 | 57.12 |
| 10 | 6 | 1 | 1 | 6 | 472.39 | 95.83% | 21.08 | 187.47 |
| 11 | 3 | 2 | 1 | 6 | 544.51 | 95.28% | 43.86 | 262.94 |
| 12 | 6 | 2 | 1 | 6 | 486.20 | 93.43% | 23.64 | 122.86 |
| 13 | 3 | 1 | 2 | 6 | 493.11 | 96.29% | 27.38 | 243.54 |
| 14 | 6 | 1 | 2 | 6 | 473.69 | 94.70% | 29.93 | 52.06 |
| 15 | 3 | 2 | 2 | 6 | 587.37 | 87.58% | 48.99 | 167.39 |
| 16 | 6 | 2 | 2 | 6 | 522.16 | 95.69% | 25.54 | 87.01 |
| 17 | 5 | 2 | 2 | 5 | 491.69 | 85.97% | 17.51 | 237.92 |
| 18 | 4 | 1 | 1 | 4 | 473.33 | 94.93% | 23.27 | 56.25 |

**Supplementary Material 5 – DoE shifts – Physicians**

Table 5 – DOE design used in Physicians

| **Run Order** | **07:00 - 19:00** | **09:00 - 21:00** | **11:00 - 23:00** | **19:00 -07:00** | **LOS** | **Treated** | **Triage-Bed** | **Prep-SSU** |
| --- | --- | --- | --- | --- | --- | --- | --- | --- |
| 1 | 3 | 0 | 0 | 3 | 2244.45 | 66.69% | 267.88 | 24.41 |
| 2 | 6 | 0 | 0 | 3 | 760.90 | 94.18% | 141.40 | 80.35 |
| 3 | 3 | 2 | 0 | 3 | 1056.99 | 87.14% | 165.61 | 58.41 |
| 4 | 6 | 2 | 0 | 3 | 515.56 | 94.57% | 39.42 | 249.99 |
| 5 | 3 | 0 | 2 | 3 | 1010.77 | 89.29% | 147.56 | 40.55 |
| 6 | 6 | 0 | 2 | 3 | 458.79 | 89.81% | 8.68 | 155.81 |
| 7 | 3 | 2 | 2 | 3 | 409.21 | 95.79% | 9.63 | 91.80 |
| 8 | 6 | 2 | 2 | 3 | 463.41 | 95.57% | 25.09 | 218.75 |
| 9 | 3 | 0 | 0 | 6 | 833.77 | 90.85% | 117.81 | 183.54 |
| 10 | 6 | 0 | 0 | 6 | 495.78 | 88.47% | 23.99 | 108.28 |
| 11 | 3 | 2 | 0 | 6 | 503.91 | 95.03% | 29.18 | 99.24 |
| 12 | 6 | 2 | 0 | 6 | 426.91 | 96.25% | 12.31 | 142.35 |
| 13 | 3 | 0 | 2 | 6 | 530.18 | 94.94% | 36.45 | 159.74 |
| 14 | 6 | 0 | 2 | 6 | 434.31 | 96.44% | 15.41 | 112.94 |
| 15 | 3 | 2 | 2 | 6 | 439.53 | 95.76% | 13.16 | 181.30 |
| 16 | 6 | 2 | 2 | 6 | 436.79 | 96.34% | 9.55 | 206.96 |
| 17 | 5 | 1 | 1 | 5 | 426.19 | 96.38% | 9.58 | 72.60 |
| 18 | 5 | 1 | 1 | 5 | 429.79 | 96.25% | 9.36 | 107.42 |

**Supplementary Material 6 – DoE shifts – Porters**

Table 6 – DOE design used in Porters

| **Run Order** | **07:00 - 19:00** | **10:00 - 18:00** | **19:00 - 07:00** | **LOS** | **Treated** | **Triage-Bed** | **Prep-SSU** |
| --- | --- | --- | --- | --- | --- | --- | --- |
| 1 | 1 | 1 | 1 | 2149.58 | 15.53% | 311.41 | 58.12 |
| 2 | 2 | 1 | 1 | 2503.46 | 50.26% | 306.18 | 47.45 |
| 3 | 1 | 2 | 1 | 2503.46 | 50.26% | 306.18 | 47.45 |
| 4 | 2 | 2 | 1 | 2095.89 | 73.21% | 211.41 | 57.18 |
| 5 | 1 | 1 | 2 | 1202.6 | 49.27% | 164.69 | 72.95 |
| 6 | 2 | 1 | 2 | 583.59 | 92.62% | 48.14 | 165.39 |
| 7 | 1 | 2 | 2 | 899.83 | 84.97% | 80.6 | 129.51 |
| 8 | 2 | 2 | 2 | 563.9 | 95.19% | 46.3 | 158.94 |
| 2 | 3 | 2 | 2 | 513.84 | 95.95% | 40.35 | 145.75 |
| 3 | 2 | 3 | 2 | 547.65 | 95.09% | 42.23 | 148.08 |
| 4 | 3 | 3 | 2 | 608.03 | 95.42% | 58.43 | 234.28 |
| 5 | 2 | 2 | 3 | 531.85 | 95.03% | 38.91 | 263.73 |
| 6 | 3 | 2 | 3 | 496.4 | 95.34% | 24.62 | 116.48 |
| 7 | 2 | 3 | 3 | 555.8 | 95.34% | 52.9 | 305.48 |
| 8 | 3 | 3 | 3 | 485.05 | 96.11% | 26.6 | 159.66 |
