# Supplementary material for: Lean thinking by integrating with discrete event simulation and design of experiments: an emergency department expansion
Source: PeerJ Comput Sci. 2020 Aug 10;6:e284. doi: 10.7717/peerj-cs.284 (PMC7924453; doi:10.7717/peerj-cs.284)
Supplement: Supplemental Information 2 [file peerj-cs-06-284-s002.docx]

### ED Data Tables

#### 1. Walk-In Arrivals

The following tables define mean arrival rates by time and day of week for the combined CBRH, NS, & NW emergency departments. Use Poisson processes to model CTAS arrivals incoming arrival rates for each day and time period, across CTAS levels.

**CTAS 01 Arrivals (mean counts)**

| CTAS 01 | Two-Hour Time Segment (notated by start hour on 24 hour clock) | | | | | | | | | | | |
| --- | --- | --- | --- | --- | --- | --- | --- | --- | --- | --- | --- | --- |
|  | 0 | 2 | 4 | 6 | 8 | 10 | 12 | 14 | 16 | 18 | 20 | 22 |
| Sun | .06 | .04 | .02 | .04 | .04 | .04 | .06 | .04 | .08 | .04 | .04 | .08 |
| Mon | .02 | .02 | .06 | .00 | .04 | .00 | .06 | .04 | .13 | .02 | .04 | .02 |
| Tues | .04 | .04 | .06 | .02 | .12 | .06 | .08 | .10 | .04 | .08 | .02 | .04 |
| Wed | .02 | .02 | .04 | .02 | .04 | .04 | .06 | .00 | .08 | .00 | .06 | .02 |
| Thurs | .04 | .04 | .00 | .04 | .06 | .13 | .00 | .08 | .04 | .02 | .06 | .04 |
| Fri | .04 | .04 | .06 | .04 | .04 | .06 | .02 | .10 | .08 | .04 | .12 | .00 |
| Sat | .02 | .04 | .00 | .08 | .00 | .06 | .02 | .06 | .00 | .06 | .04 | .04 |

Table 1.1 – CTAS1 Mean Walk-In Arrivals

**CTAS 02 Arrivals (mean counts)**

| CTAS 02 | Two-Hour Time Segment (notated by start hour on 24 hour clock) | | | | | | | | | | | |
| --- | --- | --- | --- | --- | --- | --- | --- | --- | --- | --- | --- | --- |
|  | 0 | 2 | 4 | 6 | 8 | 10 | 12 | 14 | 16 | 18 | 20 | 22 |
| Sun | 1.63 | .84 | 1.24 | 1.08 | 2.44 | 2.73 | 2.88 | 3.58 | 3.17 | 3.48 | 3.42 | 2.19 |
| Mon | 1.37 | 1.32 | 1.04 | 1.17 | 2.79 | 3.69 | 3.73 | 3.46 | 3.73 | 3.23 | 2.73 | 2.19 |
| Tues | 1.33 | 1.02 | .92 | .87 | 2.73 | 3.85 | 3.63 | 3.46 | 3.69 | 3.17 | 2.85 | 2.21 |
| Wed | 1.37 | 1.14 | .81 | 1.21 | 2.46 | 3.71 | 3.42 | 3.46 | 3.52 | 2.94 | 2.62 | 1.79 |
| Thurs | 1.08 | 1.37 | .84 | 1.17 | 3.08 | 3.56 | 3.19 | 3.77 | 3.27 | 3.08 | 2.33 | 2.10 |
| Fri | 1.40 | .70 | .98 | 1.02 | 2.65 | 3.19 | 3.73 | 3.25 | 3.60 | 3.15 | 2.67 | 1.90 |
| Sat | 1.62 | 1.08 | .98 | .94 | 2.34 | 3.25 | 3.34 | 3.51 | 3.42 | 3.15 | 2.43 | 2.28 |

Table 1.2 – CTAS2 Mean Walk-In Arrivals

**CTAS 03 Arrivals (mean counts)**

| CTAS 03 | Two-Hour Time Segment (notated by start hour on 24 hour clock) | | | | | | | | | | | |
| --- | --- | --- | --- | --- | --- | --- | --- | --- | --- | --- | --- | --- |
|  | 0 | 2 | 4 | 6 | 8 | 10 | 12 | 14 | 16 | 18 | 20 | 22 |
| Sun | 2.25 | 1.84 | 1.57 | 2.31 | 6.04 | 7.38 | 7.37 | 6.69 | 6.29 | 6.25 | 4.96 | 3.33 |
| Mon | 2.04 | 1.50 | 1.79 | 2.19 | 8.65 | 10.63 | 9.33 | 6.96 | 6.17 | 5.37 | 5.06 | 3.56 |
| Tues | 2.04 | 1.81 | 1.32 | 3.31 | 10.29 | 10.52 | 9.88 | 8.23 | 6.33 | 5.73 | 4.48 | 3.25 |
| Wed | 2.29 | 1.32 | 1.35 | 2.65 | 8.83 | 9.62 | 8.31 | 7.71 | 6.52 | 5.69 | 4.81 | 3.04 |
| Thurs | 1.90 | 1.46 | 1.04 | 1.94 | 9.23 | 9.73 | 8.85 | 8.25 | 6.17 | 5.02 | 4.23 | 3.60 |
| Fri | 1.88 | 1.50 | 1.15 | 2.83 | 8.94 | 9.87 | 9.12 | 7.73 | 6.13 | 5.77 | 4.35 | 3.71 |
| Sat | 2.04 | 1.29 | 1.48 | 2.42 | 6.81 | 8.40 | 8.25 | 6.89 | 6.13 | 5.49 | 4.75 | 3.13 |

Table 1.3 – CTAS3 Mean Walk-In Arrivals

**CTAS 04 Arrivals (mean counts)**

| CTAS 04 | Two-Hour Time Segment (notated by start hour on 24 hour clock) | | | | | | | | | | | |
| --- | --- | --- | --- | --- | --- | --- | --- | --- | --- | --- | --- | --- |
|  | 0 | 2 | 4 | 6 | 8 | 10 | 12 | 14 | 16 | 18 | 20 | 22 |
| Sun | 1.38 | .82 | 1.24 | 3.27 | 8.85 | 9.25 | 7.44 | 6.06 | 4.73 | 4.62 | 3.94 | 2.79 |
| Mon | 1.43 | 1.00 | 1.04 | 3.29 | 12.17 | 10.87 | 7.96 | 7.02 | 4.88 | 4.33 | 3.98 | 2.15 |
| Tues | 1.31 | .90 | 1.10 | 5.27 | 16.71 | 14.15 | 10.90 | 9.33 | 6.65 | 5.42 | 3.62 | 2.27 |
| Wed | 1.12 | .66 | .65 | 3.73 | 16.06 | 13.60 | 12.37 | 9.87 | 6.33 | 4.35 | 3.44 | 2.73 |
| Thurs | 1.37 | .52 | .96 | 4.02 | 15.85 | 13.83 | 10.60 | 9.90 | 6.17 | 4.38 | 3.90 | 2.60 |
| Fri | 1.46 | 1.06 | .75 | 4.15 | 13.73 | 11.50 | 9.92 | 9.10 | 5.94 | 4.85 | 3.50 | 2.90 |
| Sat | 1.45 | .88 | 1.08 | 3.45 | 11.15 | 11.17 | 9.26 | 7.34 | 5.26 | 5.08 | 3.74 | 2.60 |

Table 1.4 – CTAS4 Mean Walk-In Arrivals

**CTAS 05 Arrivals (mean counts)**

| CTAS 05 | Two-Hour Time Segment (notated by start hour on 24 hour clock) | | | | | | | | | | | |
| --- | --- | --- | --- | --- | --- | --- | --- | --- | --- | --- | --- | --- |
|  | 0 | 2 | 4 | 6 | 8 | 10 | 12 | 14 | 16 | 18 | 20 | 22 |
| Sun | .21 | .06 | .06 | .56 | 1.54 | 1.02 | .67 | .38 | .33 | .65 | .44 | .27 |
| Mon | .16 | .04 | .06 | .54 | 1.50 | 1.19 | 1.13 | .69 | .46 | .56 | .48 | .21 |
| Tues | .08 | .08 | .08 | .48 | 1.46 | 1.04 | .98 | .94 | .65 | .58 | .50 | .29 |
| Wed | .13 | .04 | .15 | .37 | 1.88 | 1.13 | .87 | .94 | .50 | .67 | .58 | .40 |
| Thurs | .14 | .15 | .10 | .38 | 1.87 | 1.08 | 1.02 | 1.15 | .79 | .38 | .33 | .31 |
| Fri | .14 | .14 | .12 | .31 | 1.37 | 1.38 | .96 | .75 | .56 | .31 | .52 | .50 |
| Sat | .19 | .12 | .06 | .58 | 1.58 | 1.04 | .79 | .58 | .36 | .49 | .45 | .26 |

Table 1.5 – CTAS5 Mean Walk-In Arrivals

#### 2. EHS Arrivals

The following table defines EHS mean arrivals by day and time. Define Poisson distributions for EHS arrivals as you have for walk-in arrivals.

**CTAS 01 EHS Arrivals (mean counts)**

| CTAS 01 | Two-Hour Time Segment (notated by start hour on 24 hour clock) | | | | | | | | | | | |
| --- | --- | --- | --- | --- | --- | --- | --- | --- | --- | --- | --- | --- |
|  | 0 | 2 | 4 | 6 | 8 | 10 | 12 | 14 | 16 | 18 | 20 | 22 |
| Sun | 0.02 | 0.04 | 0.02 | 0.02 | 0.02 | 0.04 | 0.06 | 0.04 | 0.08 | 0.02 | 0.04 | 0.06 |
| Mon | 0.02 | 0.02 | 0.06 | 0 | 0.04 | 0 | 0.04 | 0.02 | 0.1 | 0 | 0.04 | 0.02 |
| Tues | 0.04 | 0.04 | 0.04 | 0.02 | 0.08 | 0.06 | 0.08 | 0.08 | 0.04 | 0.08 | 0.02 | 0.04 |
| Wed | 0.02 | 0.02 | 0.04 | 0.02 | 0.04 | 0.04 | 0.06 | 0 | 0.08 | 0 | 0.04 | 0 |
| Thurs | 0.04 | 0.02 | 0 | 0.02 | 0.04 | 0.13 | 0 | 0.06 | 0.02 | 0.02 | 0.04 | 0.04 |
| Fri | 0.04 | 0.02 | 0.06 | 0.04 | 0.04 | 0.04 | 0.02 | 0.1 | 0.06 | 0.04 | 0.08 | 0 |
| Sat | 0.02 | 0.04 | 0 | 0.06 | 0 | 0.04 | 0.02 | 0.04 | 0 | 0.06 | 0.04 | 0.04 |

Table 2.1 – CTAS1 Mean EHS Arrivals

**CTAS 02 EHS Arrivals (mean counts)**

| CTAS 02 | Two-Hour Time Segment (notated by start hour on 24 hour clock) | | | | | | | | | | | |
| --- | --- | --- | --- | --- | --- | --- | --- | --- | --- | --- | --- | --- |
|  | 0 | 2 | 4 | 6 | 8 | 10 | 12 | 14 | 16 | 18 | 20 | 22 |
| Sun | 0.6 | 0.29 | 0.37 | 0.31 | 0.62 | 0.81 | 0.79 | 1 | 1.04 | 1.21 | 1.08 | 0.56 |
| Mon | 0.51 | 0.5 | 0.31 | 0.31 | 0.85 | 0.62 | 0.96 | 0.85 | 1.15 | 0.81 | 0.83 | 0.62 |
| Tues | 0.37 | 0.38 | 0.28 | 0.15 | 0.58 | 1 | 0.96 | 1.25 | 1.08 | 0.67 | 0.98 | 0.73 |
| Wed | 0.58 | 0.46 | 0.33 | 0.46 | 0.6 | 0.9 | 0.9 | 0.77 | 1 | 0.92 | 0.77 | 0.54 |
| Thurs | 0.35 | 0.52 | 0.33 | 0.37 | 0.85 | 0.69 | 0.81 | 0.9 | 0.88 | 0.98 | 0.63 | 0.69 |
| Fri | 0.42 | 0.16 | 0.38 | 0.27 | 0.63 | 0.87 | 0.81 | 1.1 | 0.96 | 1.08 | 0.77 | 0.73 |
| Sat | 0.75 | 0.39 | 0.42 | 0.25 | 0.58 | 0.91 | 1.02 | 1.08 | 1.21 | 0.98 | 0.83 | 0.75 |

Table 2.2 – CTAS2 Mean EHS Arrivals

**CTAS 03 EHS Arrivals (mean counts)**

| CTAS 03 | Two-Hour Time Segment (notated by start hour on 24 hour clock) | | | | | | | | | | | |
| --- | --- | --- | --- | --- | --- | --- | --- | --- | --- | --- | --- | --- |
|  | 0 | 2 | 4 | 6 | 8 | 10 | 12 | 14 | 16 | 18 | 20 | 22 |
| Sun | 0.83 | 0.61 | 0.39 | 0.46 | 0.94 | 1.1 | 1.06 | 1 | 1.15 | 1.13 | 0.6 | 0.77 |
| Mon | 0.55 | 0.36 | 0.54 | 0.37 | 0.88 | 1.21 | 1.38 | 1.15 | 1.08 | 0.85 | 0.96 | 0.73 |
| Tues | 0.51 | 0.46 | 0.44 | 0.58 | 1.15 | 1.5 | 1.65 | 1.23 | 1.27 | 1.17 | 1.06 | 0.85 |
| Wed | 0.75 | 0.32 | 0.38 | 0.42 | 0.85 | 1.17 | 1.33 | 1.29 | 1.37 | 1.17 | 1.37 | 0.85 |
| Thurs | 0.35 | 0.43 | 0.27 | 0.29 | 0.9 | 1.33 | 1.37 | 1.25 | 1.21 | 0.92 | 0.87 | 0.56 |
| Fri | 0.54 | 0.52 | 0.27 | 0.46 | 0.98 | 1.46 | 1.23 | 1.21 | 1.46 | 1.15 | 0.85 | 0.83 |
| Sat | 0.68 | 0.31 | 0.42 | 0.49 | 0.85 | 1.34 | 1.62 | 0.7 | 1.19 | 1.11 | 1 | 0.68 |

Table 2.3 – CTAS3 Mean EHS Arrivals

**CTAS 04 EHS Arrivals (mean counts)**

| CTAS 04 | Two-Hour Time Segment (notated by start hour on 24 hour clock) | | | | | | | | | | | |
| --- | --- | --- | --- | --- | --- | --- | --- | --- | --- | --- | --- | --- |
|  | 0 | 2 | 4 | 6 | 8 | 10 | 12 | 14 | 16 | 18 | 20 | 22 |
| Sun | 0.13 | 0.16 | 0.12 | 0.17 | 0.35 | 0.46 | 0.37 | 0.23 | 0.38 | 0.37 | 0.31 | 0.29 |
| Mon | 0.22 | 0.18 | 0.13 | 0.12 | 0.38 | 0.48 | 0.4 | 0.37 | 0.35 | 0.19 | 0.29 | 0.12 |
| Tues | 0.2 | 0.15 | 0.14 | 0.19 | 0.33 | 0.75 | 0.46 | 0.23 | 0.46 | 0.33 | 0.25 | 0.23 |
| Wed | 0.12 | 0 | 0.04 | 0.12 | 0.33 | 0.44 | 0.63 | 0.37 | 0.35 | 0.17 | 0.21 | 0.21 |
| Thurs | 0.27 | 0.04 | 0.12 | 0.12 | 0.27 | 0.54 | 0.31 | 0.4 | 0.19 | 0.31 | 0.42 | 0.29 |
| Fri | 0.2 | 0.2 | 0.04 | 0.12 | 0.35 | 0.62 | 0.63 | 0.46 | 0.44 | 0.29 | 0.29 | 0.29 |
| Sat | 0.17 | 0.16 | 0.1 | 0.11 | 0.4 | 0.43 | 0.32 | 0.3 | 0.36 | 0.42 | 0.36 | 0.21 |

Table 2.4 – CTAS4 Mean EHS Arrivals

**CTAS 05 EHS Arrivals (mean counts)**

| CTAS 05 | Two-Hour Time Segment (notated by start hour on 24 hour clock) | | | | | | | | | | | |
| --- | --- | --- | --- | --- | --- | --- | --- | --- | --- | --- | --- | --- |
|  | 0 | 2 | 4 | 6 | 8 | 10 | 12 | 14 | 16 | 18 | 20 | 22 |
| Sun | 0 | 0.02 | 0 | 0 | 0.02 | 0.02 | 0.02 | 0.02 | 0.04 | 0.04 | 0 | 0.02 |
| Mon | 0.02 | 0 | 0.02 | 0 | 0.04 | 0.08 | 0.06 | 0 | 0.04 | 0 | 0.04 | 0 |
| Tues | 0 | 0 | 0 | 0.04 | 0.04 | 0 | 0 | 0.06 | 0.04 | 0.02 | 0 | 0 |
| Wed | 0.02 | 0 | 0 | 0 | 0.08 | 0.04 | 0.02 | 0.02 | 0.02 | 0 | 0.02 | 0 |
| Thurs | 0 | 0 | 0 | 0.02 | 0.02 | 0 | 0.04 | 0.04 | 0.04 | 0.04 | 0.04 | 0.02 |
| Fri | 0 | 0.02 | 0.02 | 0.02 | 0.04 | 0.04 | 0.02 | 0.04 | 0.04 | 0.04 | 0.02 | 0 |
| Sat | 0.04 | 0 | 0 | 0 | 0.06 | 0 | 0.02 | 0.02 | 0.02 | 0 | 0 | 0 |

Table 2.5 – CTAS5 Mean EHS Arrivals
