# Supplementary material for: Lean thinking by integrating with discrete event simulation and design of experiments: an emergency department expansion
Source: PeerJ Comput Sci. 2020 Aug 10;6:e284. doi: 10.7717/peerj-cs.284 (PMC7924453; doi:10.7717/peerj-cs.284)
Supplement: Supplemental Information 5 [file peerj-cs-06-284-s005.docx]

**Instruction to open code in FlexSim HC**

In both models (current and future state), you should use FlexSim HC 5.3.10 version.

In the bottom of the software, some field were programed:

- Flowchart: it is possible to verify the patient flow to the hospital. Arrows indicate where the patient can go.
- Patient Tracks: the patient flow is programmed in this field. If you open WICTAS1, you see all the possibilities for this type of patient. In “Arrival” we programmed how the patient arrives in the model and the destination. The time for each process is completed in the “Processing Time” field (Table A1) data provided in Table A. Figure 1 shows how we have programmed.


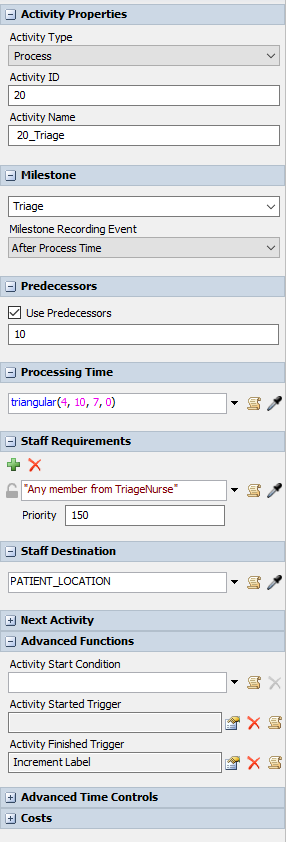


Figure 1 – Fields to complete in the process

- PCIs: indicates the patient visual.
- Doors: If you click in the “door” in the model screen, it will open a window with the arrival rate in a table (data provided in Supplemental Material 2). Figure 2 shows the Table.


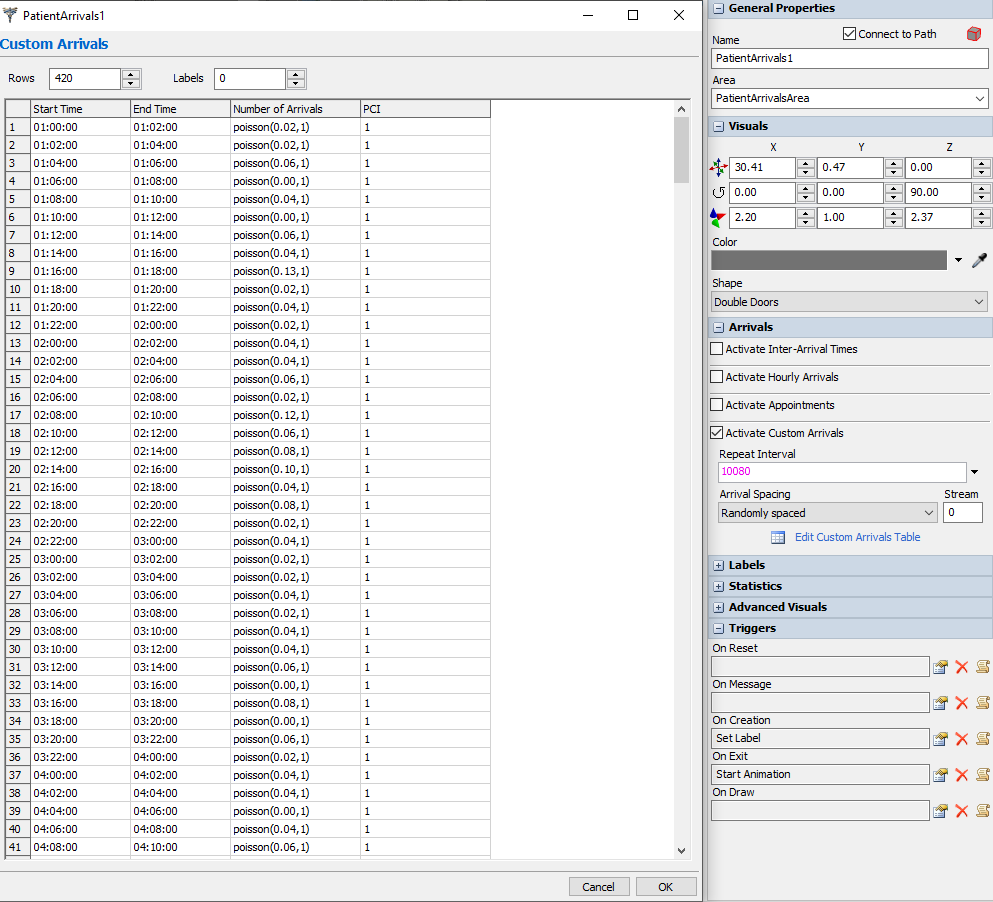


Figure 2 – Arrival rate Table

- Experimenter: you can make the scenarios in this field and run the simulation. In Dashboard you can see the answers for the experimentation (Supplemental Material 1).
